# Surgical Updates in the Treatment of Pelvic Organ Prolapse

**DOI:** 10.5041/RMMJ.10294

**Published:** 2017-04-28

**Authors:** Julia Geynisman-Tan, Kimberly Kenton

**Affiliations:** Division of Female Pelvic Medicine and Reconstructive Surgery, Northwestern University, Chicago, Illinois, USA

**Keywords:** Apical suspension, pelvic organ prolapse, sacrocolpopexy, stress incontinence, uterine preservation, vaginal mesh

## Abstract

Pelvic organ prolapse affects approximately 8% of women, and the demand for pelvic organ prolapse surgery is expected to increase by nearly 50% over the next 40 years. The surgical techniques used to correct pelvic organ prolapse have evolved over the last 10 years, with multiple well-designed studies addressing the risks, outcomes, reoperation rates, and optimal surgical approaches. Here we review the most recent evidence on the route of access, concomitant procedures, and synthetic materials for augmenting the repair. Ultimately, this review highlights that there is no optimal method for correcting pelvic organ prolapse and that the risks, benefits, and approaches should be discussed in a patient-centered, goal-oriented approach to decision-making.

## INTRODUCTION

Pelvic organ prolapse (POP) is a progressive herniation of the pelvic organs through the urogenital diaphragm that most commonly leads to symptoms of a vaginal bulge.^1^,^2^ Women with prolapse beyond the hymen may also report lower urinary tract (incontinence, urgency-frequency, or voiding difficulty) and bowel (obstructed defecation, fecal incontinence) symptoms. The estimated prevalence of POP is between 2.9% and 8% of the female population,^3^–^5^ and recent estimates suggest women have a 12.6% lifetime risk of undergoing surgery for prolapse.^6^ Approximately 300,000 women in the United States undergo surgical procedures for prolapse each year.^7^

Despite the prevalence and social burden of this disorder, researchers and surgeons are still waiting for a universally successful and risk-minimizing solution to this disorder. A variety of surgical approaches have been developed and optimized over the last half century to enhance durability, minimize risks, and shorten recovery. However, the treatment of prolapse remains a matter of individualizing patient outcome goals while navigating the risks of hernia recurrence, surgical complications, and mesh complications. Therefore, the objectives of this paper are to highlight some best practices that have emerged over the last decade and to expose several issues where our data are inconclusive and warrant further investigation.

## ALL ABOUT THE APEX

Perhaps the most important development in prolapse surgery over the last decade is the determination that apical support is the key to a successful prolapse repair.^8^ Multiple studies demonstrate that the apex descends with the anterior compartment and that correction of the anterior wall without addressing the apex increases the risk of recurrent prolapse.^8^–^10^ In a paper by Rooney et al. in which they studied the relationship between anterior and apical prolapse using the pelvic organ prolapse quantification system (POP-Q),^11^ the investigators found a linear correlation between the positions of the apex (POP-Q point C) and the most prolapsed portion of the anterior vaginal wall (POP-Q point Ba).^9^ When the anterior vaginal wall prolapses to the level of the hymen (point Ba=0)—the point at which most women will become symptomatic—the apex is approximately 4.5 cm inside the hymen.^9^ In other words, when a woman has clinically significant prolapse to the hymen, she will have nearly 5 cm of apical support loss. This physical exam finding was confirmed radiologically in several studies by DeLancey’s team.^8^,^10^ On MRI modeling of pelvic organ descent with Valsalva, Chen et al. showed that a 90% impairment of apical support led to an increase in anterior wall prolapse from 0.3 cm to 1.9 cm.^10^ Similarly, using MRI, the same group showed that 77% of anterior vaginal wall descent was explained by the position of the apex and length of the anterior vaginal wall.^12^ These studies supplement a growing body of literature on the importance of apical (cardinal and uterosacral ligament) support in the natural history of POP.^13^,^14^

Unfortunately, not all surgeons perform this crucial apical repair at the time of POP surgery. In a review of the treatment for POP offered by physicians at a single center in the United States, Alas et al. found that, of 21 hysterectomies performed for the indication POP, only 48% included a vault suspension.^15^ Likewise, in a review of the National Hospital Discharge survey from 1979 to 2009, Stewart and colleagues found that, although there was a significant decrease in the frequency of isolated cystocele/rectocele repair procedures performed for pelvic organ prolapse, 87% of all anterior, apical, and posterior compartment prolapses were still being managed with cystocele/rectocele repair alone.^16^ Northington et al. identified women undergoing prolapse surgery with a primary diagnosis of cystocele in 2011 from the Nationwide Inpatient Sample.^17^ Only 32% of these women underwent a concomitant apical prolapse procedure, suggesting an ongoing discrepancy between evidence-based medicine and adoption into clinical practice.^17^ These data are concerning given that an unacceptably high number (17%) of women who underwent a procedure for POP will undergo a second surgery for recurrent POP within 10 years.^18^

## ROLE FOR CONCOMITANT ANTERIOR SUPPORT PROCEDURES

Performing an adequate apical repair frequently obviates the need for a concomitant repair of the anterior or posterior compartment, particularly when performing a sacrocolpopexy in which the mesh arms are attached to the anterior and posterior vagina. In a series of women with ≥stage II prolapse who underwent sacrocolpopexy without concomitant anterior or posterior repair, Guiahi et al. reported significant improvement in the most prolapsed points on the anterior (Ba) and posterior (Bp) vaginal walls 1 year after surgery.^19^ Before surgery, mean point Ba was 3.5 cm outside the hymen, and point Bp was 1 cm outside the hymen. One year after surgery, both Ba and Bp were well-supported 2 cm inside the hymen.^19^ The effect of sacrocolpopexy alone on anterior wall support is further demonstrated in the Colpopexy and Urinary Reduction Efforts (CARE) trial. In the CARE trial, continent women with ≥stage II prolapse under-going open sacrocolpopexy were randomized to receive a concomitant Burch procedure or no Burch.^20^ Women were stratified prior to the start of the trial at each study surgeon’s discretion by whether they would have a concomitant paravaginal repair to reattach the lateral vagina to the pelvic sidewall, so approximately equal numbers of women in both arms underwent paravaginal repair. There was no clinically meaningful difference in prolapse outcomes between the groups at any time point—3 months, 1 year, or 2 years.^21^, ^22^

## ROLE FOR POSTERIOR REPAIR: WHAT ARE WE PLICATING?

Just as with the anterior compartment, restoration of apical support frequently corrects posterior compartment prolapse. The CARE trial allowed performance of a posterior colporrhaphy or perineor-rhaphy at the surgeon’s discretion. In a 1-year analysis of bowel symptoms in women enrolled in the CARE trial, Bradley et al. reported on the symptomatic outcomes of 87 women who under-went concomitant posterior procedures compared to those who did not.^23^ Baseline posterior vaginal wall prolapse was not different between the groups, although obstructive defecation symptoms were slightly higher in the posterior repair group.^23^ Both groups reported significant improvement in bowel symptoms, including constipation, incomplete emptying, and pain and irritation with defecation, and had significant improvement in posterior wall vaginal outcomes; however, the women who had posterior repair reported higher rates of pain with defecation and more fecal incontinence.^23^

In an analysis of 5-year CARE posterior compartment outcomes, Grimes found that reoperation for posterior vaginal wall prolapse was more frequent in those women who had a posterior repair at the time of initial surgery. Ninety-six percent of women with posterior vaginal wall prolapse at or beyond the hymen (Ap≥0) who did not undergo a posterior repair had sustained resolution of their posterior prolapse, and none underwent posterior repair; in contrast, 12% of those who had a posterior repair had recurrent/persistent posterior prolapse, and 14% had another posterior repair.^24^ Likewise, in the Guiahi series of women who underwent sacrocolpopexy without posterior repair only 8% of women had ≥stage II posterior prolapse 1 year after sacrocolpopexy in contrast to 61% prior to sacrocolpopexy.^19^ The most prolapsed portion of the posterior vaginal wall (Bp) improved significantly after sacrocolpopexy, and the genital hiatus size decreased by approximately 1 cm.^19^

Recent data have reopened the debate on whether a rectovaginal septum even exists and whether there is any anatomic basis for posterior colporrhaphy. In an abstract presented at the 37th meeting of the American Urogynecologic Society, Maldonado et al. presented the results of an anatomic dissection of 10 cadavers.^25^ In the rectovaginal space spanning the length of the vagina from the peritoneal reflection to the perineal body, there was no distinct tissue layer visible by gross examination. On microscopic examination, there was a loose layer of fibroadipose tissue noted at the midvagina but no fibroconnective tissue along the length of the posterior vagina. The anatomist concluded that in the middle segment of the posterior vagina the fibromuscular tissue that is sometimes plicated is either split from the posterior vaginal wall or the smooth muscle of the anal canal.^25^ This study contradicts prior literature in women, which supports the embryological and functional presence of the rectovaginal septum.^26^,^27^ The definitive presence or absence of this structure could help explain the variable outcomes seen after posterior colporrhaphy. In a prospective study of 66 women under-going defect-specific posterior colporrhaphy, Kenton et al. found that 1 year after repair only 54% had resolution of difficult defecation, 43% had resolution of constipation, and 36% had resolution of manual evacuation.^28^ Finally, the subjective and objective cure of posterior compartment prolapse seems to decrease quickly after posterior colporrhaphy. In a prospective study by Maher et al. the subjective success rate following midline rectovaginal fascia plication fell from 97% at 12 months to 89% at 24 months.^29^ The objective success rate likewise fell from 87% at 12 months to 79% at 24 months.^29^ Some surgeons hypothesize that the recurrence of posterior prolapse is the result of ineffective plication of layers of the vagina without structural integrity. Therefore, further histological study on the rectovaginal septum should be pursued.

## UTERINE PRESERVATION WITH POP

With increasing evidence that apical support is the cornerstone of prolapse surgery, the focus is shifting to identifying the optimal procedure to suspend the apex. One factor in this discussion is the role of hysterectomy at the time of apical prolapse repair and how patients and physicians may perceive concomitant hysterectomy differently.^30^ With growing emphasis on patient-centered medicine and patient perception of complications and success, the treatment options offered to each woman suffering from POP should be tailored to her values.^30^,^31^

One value that women increasingly favor is uterine preservation. When presented with the choice to undergo a hysterectomy at the time of POP surgery, 36%–60% of women presenting for POP care would decline a hysterectomy.^32^,^33^ In one study, 21% of women continued to favor uterine preservation even when counseled that POP surgical outcomes may be less efficacious.^33^ Although hysterectomy is done routinely with POP repair, there are few data to support it. Some experts even argue that disruption of the uterosacral/cardinal ligament complex may weaken the pelvic floor supports even further. Others cite emerging data that transection of the utero-ovarian ligament during hysterectomy may compromise blood flow to the ovary and accelerate menopause.^34^,^35^

While the majority of high-quality long-term outcome data after prolapse surgery are in women who have undergone a hysterectomy, short-term data on outcomes of uterine-sparing POP repair are promising. Numerous studies show short-term safety and efficacy; however, these papers tend to be of low quality, including case series or cohort studies.^36^ Few well-done randomized controlled trials on this topic exist, and there are likely differences in outcomes between types of hysteropexy (vaginal versus abdominal, with and without mesh).

Retrospective comparative studies suggest sacro-spinous hysteropexy is as effective as vaginal hysterectomy with apical repair, and meta-analyses show reduced operating time, blood loss, and recovery time after sacrospinous hysteropexy.^36^ A multi-center, randomized trial comparing vaginal hysterectomy with uterosacral ligament suspension and sacrospinous hysteropexy found that sacrospinous hysteropexy was not inferior to vaginal hysterectomy with uterosacral ligament suspension for failure at the apex (≥stage II apical prolapse with bothersome symptoms or recurrent surgery) 12 months after surgery.^37^ However, 47% (47/101) of women in the sacrospinous hysteropexy group met the authors’ criteria for anatomic failure of the anterior vaginal wall compared to only 33% (33/99) in the vaginal hysterectomy arm.^37^ Likewise, in 2001, Maher et al. reported on a cohort of 43 women who underwent laparoscopic uterosacral ligament hysteropexy.^38^ At 1 year, 81% of women reported subjective cure, and 79% had no evidence of recurrent prolapse on exam.^38^ Two women in the cohort underwent a subsequent pregnancy and had no recurrence of prolapse.

One trial compared vaginal hysterectomy with uterosacral ligament suspension to open sacrohysteropexy with mesh and found similarly high apical success rates (95%) and subjective outcomes between the groups; however, the women in the sacrohysteropexy arm were more likely to undergo reoperation in the first year after surgery.^39^ In a large retrospective cohort study of 507 women who underwent laparoscopic sacrohysteropexy over a 10-year period, 94% of women reported being “very much” or “much” better, and only 2.8% underwent repeat surgery for apical prolapse.^40^ A recently published multicenter parallel cohort study compared 1-year outcomes after vaginal mesh hysteropexy (UPHOLD™, Boston Scientific, Marlborough, MA, USA) and laparoscopic sacral hysteropexy in women with stage II–IV anterior and apical prolapse who desired uterine preservation.^41^ To be included, women must have anterior vaginal wall prolapse to or beyond the hymen (POP-Q Ba≥0); apical prolapse to at least the midvagina (C≥½ total vaginal length [TVL]); and symptoms of a vaginal bulge. Investigators reported high satisfaction rates in both arms (95%) and anatomic cure rates (no reoperation or pessary use, no apical prolapse >TVL/2, and no anterior or posterior vaginal wall prolapse at or beyond the hymen) of 77%–80%.^41^ Three women in the vaginal hysteropexy arm under-went reoperation for prolapse or cervical elongation in the first year after surgery.^41^ To further study whether a hysterectomy is necessary for repair of POP, the Pelvic Floor Disorders Network designed the SUPeR trial, a multicenter, single-blinded trial which randomizes women to a vaginal hysterectomy with uterosacral ligament suspension versus an UPHOLD™ vaginal mesh hysteropexy.^42^ The results of the trial are expected in 2018, when the 3-year follow-up concludes. The UPHOLD™ vaginal support system (Boston Scientific) uses a soft polypropylene mesh with a bilateral sacrospinous suspension to improve anterior and apical prolapse. The UPHOLD™ system can be used with the uterus *in situ* in women with uterovaginal prolapse, potentially decreasing surgical time and complications associated with hysterectomy, while also avoiding deviating or narrowing of the vagina.^43^ In a prospective cohort study of 99 women undergoing UPHOLD™ vaginal mesh hysteropexy for ≥stage II prolapse investigators reported a 97.7% anterior vaginal wall success rate (POP-Q Ba<–1 and a negative response to: “Do you experience the feeling of bulging or protrusion in the vaginal area?”) and a 96.6% apical success rate (C<–½ TVL and negative response to: “Do you experience the feeling of bulging or protrusion in the vaginal area?”).^44^ A primary limitation of this study is inclusion of women with minimal or no apical prolapse (range of baseline POP-Q C measurements –9 to +9) rather than using separate predefined inclusion criteria for the apex and anterior vaginal wall. Therefore, some of the women reported as “apical successes” were cured prior to surgery, skewing the outcomes. Posterior landmarks were not reported secondary to high rates of concomitant posterior colporrhaphies. Seven women (6.5%) had mesh extrusion, one needing surgical excision in the operating room.^44^

Additional factors must be considered when planning uterine-sparing prolapse surgery. Women with a history of cervical pathology or abnormal uterine bleeding may not be good candidates for uterine preservation. Likewise, women who are at high risk of uterine malignancy due to family history or comorbidities may not be well served with hysterectomy as part of their prolapse repair. Uterine preservation necessitates ongoing surveil-lance for cervical and uterine malignancies, which may be more technically challenging with the uterus suspended or deviated following a repair.

## WHERE DO WE BEGIN?

Fortunately, there are mounting comparative data investigating the outcomes, risks, and costs associated with vaginal, laparoscopic, robotic, and open abdominal techniques to repair pelvic organ prolapse. The first step in the algorithm is deciding between abdominal and vaginal routes. Historically, abdominal surgery for prolapse centers on sacrocolpopexy with a synthetic graft material, and vaginal surgery centers on native tissue repairs suspending the apex to the sacrospinous ligament or uterosacral ligaments. In older randomized trials and a Cochrane Review comparing open sacrocolpopexy to vaginal sacrospinous ligament suspension, sacrocolpopexy was associated with better anatomic outcomes, fewer prolapse recurrences, and longer time to prolapse recurrence.^45^ In a meta-analysis comparing sacrocolpopexy with native tissue vaginal repairs, Siddiqui et al. analyzed 34 studies, which reported on “anatomic success” in women with at least 6 months of follow-up.^46^ They found that when defining success as any prolapse <stage 2 or above the hymen the pooled odds ratio for anatomic success was 2.04 with mesh sacrocolpopexy compared to native tissue repair. However, there were more adverse events, including small-bowel obstruction and mesh erosion, with sacrocolpopexy. They conclude that when durability is the greatest priority for the patient, sacrocolpopexy may be preferred.^46^ However, for many patients, durability may not be the most important factor. For patients with medical or surgical comorbidities the risks of an abdominal approach may outweigh the benefits of durability. Unfortunately, no randomized trial of uterosacral ligament suspension versus sacrocolpopexy exists.

Although the majority of data on sacrocolpopexy outcomes compare vaginal repairs with an open sacrocolpopexy technique, open abdominal sacrocolpopexy is no longer recommended as the first-line approach to sacrocolpopexy, with the exception of rare and carefully selected cases. Across surgical subspecialties, open abdominal techniques are associated with longer hospitalizations, increased pain, higher costs, and greater wound complications.^47^,^48^ This would be tolerable if open approaches led to better outcomes. However, in a 2013 multi-center, randomized equivalence trial comparing open versus laparoscopic sacrocolpopexy at 1 year, investigators in the United Kingdom found that apical prolapse outcomes (POP-Q point C) and patients’ perception of improvement on the Patient Global Impression of Improvement scale were equivalent.^49^ Similarly, there was no difference in surgical complications or mesh exposures between the groups. However, drop in hemoglobin and length of stay were significantly lower in the laparoscopic arm, suggesting that laparoscopic sacrocolpopexy may have benefits over open techniques.^49^

If the patient selects a native tissue vaginal repair to avoid the complications of mesh, the two best-studied procedures are sacrospinous ligament suspension and uterosacral ligament suspension. Numerous approaches have been described for sacrospinous ligament suspension, including posterior, apical, and anterior approaches and unilateral or bilateral suspension. A commonly used technique, the Michigan four-wall sacrospinous ligament suspension, accesses the ligament via an apical approach and attaches both the anterior and posterior vaginal walls directly to the sacrospinous ligament.^50^ It differs from traditional sacrospinous ligament suspension, in which the ligament is accessed via a posterior approach and only attaches the upper aspect of the right posterior vaginal wall to the ligament,^51^ theoretically predisposing it to anterior vaginal wall recurrence. In a recent review of the long-term outcomes (>5 years) of the Michigan modification of the sacrospinous ligament suspension, the majority (90%) of patients were satisfied up to 8 years after the surgery.^52^ Similarly high outcomes are reported for uterosacral ligament suspensions. Uterosacral ligament suspension was reintroduced by Dr Bob Shull in 2000 and gained in popularity.^53^ In a review of 5-year outcomes of uterosacral ligament suspension performed on 72 women at a single center, surgical failure (defined as symptomatic recurrent prolapse of stage 2 or greater in one or more segments) occurred in only 15% of patients.^54^ As both types of native tissue repair have favorable outcomes without the risk of mesh complications, the two procedures were compared in a head-to-head trial.^55^ The OPTIMAL trial was a randomized trial comparing uterosacral ligament suspension to sacrospinous ligament suspension with a primary outcome defined as a composite outcome of surgical success. The composite was defined by three measures: (1) *anatomic success*: no apical descent greater than one-third into the vaginal canal or anterior *or* posterior vaginal wall beyond the hymen; (2) no bothersome vaginal bulge symptoms; and (3) no re-treatment for prolapse at 2 years. At 2 years, there was no statistically significant difference in surgical success between the surgical groups (uterosacral ligament suspension [USLS] 59.2% versus sacrospinous ligament fixation [SSLF] 60.5%) and no clinically significant differences in any of the primary outcome components.^55^ There was a low rate of adverse events that was not significantly different between groups. Overall, 18.0% of women (55/305) developed bothersome vaginal bulge symptoms, 17.5% (54/308) had anterior or posterior prolapse, or both, beyond the hymen, and 5.1% (16/316) underwent either conservative or surgical retreatment by 2 years.^55^ The proportion of women with recurrent anterior (USLS 15.5% versus SSLF 13.7%) or posterior prolapse (USLS 4.5% versus SSLF 7.2%) beyond the hymen was not significantly different between treatment groups.^55^

When considering the *tools to use* for minimally invasive abdominal approaches, the literature is clearer on the benefits of laparoscopic over robotic surgical systems. In one blinded, randomized trial, Paraiso et al. assigned post-hysterectomy vaginal vault prolapse patients to laparoscopic versus robotic sacrocolpopexy.^56^ Anesthesia time, total suturing time, total operating room time, and cost were all higher in the robotic group, without any differences in vaginal support or functional outcomes at 1 year.^56^ This was confirmed by a multicenter randomized trial of laparoscopic versus robotic sacrocolpopexy performed by Anger et al., which also found increased hospital costs, longer operating room times, and higher pain scores in the robotic arm, without any differences in symptom bother, POP stage at 6 months, or adverse events.^57^ A planned ancillary study of the Anger trial comparing the wound healing of laparoscopic versus robotic port site incisions showed an improved cosmetic appearance from laparoscopic incisions over robotic.^58^ However, laparoscopic skills are known to require a steeper, more difficult, learning curve and skilled assistants. For this reason, robotic sacrocolpopexy has been adopted by many surgeons who wish to offer minimally invasive repairs with a faster and easier learning curve and improved optics. In a meta-analysis of the outcomes following robotic sacrocolpopexy, Hudson et al. found that this procedure led to a 98.6% success rate (defined as apical prolapse less than or equal to stage 1) with a 2% cystotomy rate and a 4.1% mesh erosion rate.^59^ They also reported a 3.3% reoperation rate for prolapse and a 6.6% total reoperation rate.^59^ The conclusion to draw from these and similar studies is that the counseling around the route of access must be goal-centered and driven by the patient’s priorities as they intersect with the surgeon’s experience.

## THE MESH OF PROS AND CONS

A review on the surgical updates in pelvic organ prolapse is not complete without a brief discussion of mesh. Mesh implants play an important role in pelvic organ prolapse repair. Considering that 30% of women will have a second operation for POP, a material that may reduce that risk of recurrence is desirable.^60^ However, as described above, the data on whether mesh-augmented surgery is superior to native tissue repairs are variable and likely dependent on route of surgery (abdominal or vaginal) and type of mesh placement.

As mentioned above, older randomized trials and a Cochrane Review comparing abdominally placed mesh (i.e. sacrocolpopexy) to vaginal sacrospinous ligament suspension found sacrocolpopexy to be associated with better anatomic outcomes, fewer prolapse recurrences, and longer time to prolapse recurrence.^45^,^61^,^62^ In 2004 Maher et al. found no significant differences in all-cause reoperation between mesh-augmented abdominal sacrocolpopexy (13%) versus sacrospinous ligament fixation (16%).^61^ Subjective cure rates were high (94% and 91%) after mesh sacrocolpopexy and native tissue repair; how-ever, 19% of women in the native tissue group had apical prolapse to the hymen 6 months after surgery compared to 4% after sacrocolpopexy.^61^ Another randomized controlled trial comparing abdominal sacrocolpopexy (with the cervix *in situ*) to bilateral sacrospinous vault suspension, by Benson et al., reported a reoperation rate of 33% in the vaginal group and 16% in the abdominal group at a mean of 2.5 years of follow-up.^62^ This difference in reoperation rates is further elucidated in the 2008 retrospective cohort study by Thompson et al. in which subjects who underwent an abdominal sacrocolpopexy had a 3% rate of reoperation (all for mesh removal) versus a 33% reoperation rate in the uterosacral ligament suspension group (all for recurrent prolapse).^63^

While it seems that abdominally placed mesh for POP may reduce the risk of recurrence and reoperation for POP, it is associated with a non-negligible risk of vaginal mesh exposures and/or erosions into bladder or bowel. Although we continue to search for the ideal synthetic or biologic graft material, modern synthetic POP meshes possess improved qualities compared to older synthetic meshes. Mesh exposure/erosion rates after sacrocolpopexy range from 2% to 10% overall, and increase to 23%–40% when the mesh is attached transvaginally after a total vaginal hysterectomy.^64^–^66^ However, mesh complication data from earlier studies may not be generalizable to current meshes, which are lighter and more porous. For example, in the CARE trial, which reported mesh exposure/erosion rates as high as 10.5% by 7 years, more than 50% of the women did not have a type 1 polypropylene mesh used in their prolapse repair.^67^ Earlier-generation type III and IV meshes, such as GORE-TEX or MERSILENE, are associated with higher complication rates.^64^ Similarly, although studies report that total hysterectomy at the time of sacrocolpopexy increases the rates of mesh erosion, when considering only studies in which type-I polypropylene mesh was used, no increased risk of mesh erosion with concomitant total hysterectomy is reported.^68^–^71^

In addition, the type of suture used to affix the mesh can contribute to the erosion rate. Historically, open abdominal sacrocolpopexies were performed with permanent suture, but many surgeons have converted to delayed absorbable suture in the last decade. In a retrospective study comparing delayed absorbable monofilament suture to permanent suture, absorbable suture was associated with a reduced erosion rate (0% versus 3.7%).^72^ The hypothesis that suture type leads to erosion is currently being tested in a multicenter randomized controlled trial of permanent monofilament suture (GORE-TEX^®^ Flagstaff, AZ, USA) compared to a monofilament delayed absorbable suture (polydioxanone) for vaginal attachment of the mesh. Regardless of the results, this study is the natural next step in the ever-evolving pursuit of the “gold standard” hernia repair.

More recently, in an attempt to combine the outcome benefits of abdominally placed synthetic meshes with the quicker recovery and fewer complications of vaginal surgery, transvaginally placed synthetic meshes for POP have gained popularity. Since the first Food and Drug Administration safety communication regarding transvaginal mesh in 2008,^73^ the number of studies on transvaginal mesh has increased exponentially. Most outcome data on transvaginal mesh placement report outcomes based on vaginal compartment treated with mesh, i.e. posterior, anterior, and apical prolapse.

The majority of evidence in support of transvaginal mesh is for anterior compartment POP. Altman et al. randomized 389 women with ≥stage II anterior vaginal wall prolapse to traditional anterior colporrhaphy or trocar-guided placement of polypropylene mesh.^74^ Transvaginal mesh was superior to native tissue repair in anatomic and subjective cure at 1 year, with composite success rates of 61% for transvaginal mesh and 35% for native tissue repair; however, the improved success was associated with higher rates of adverse events, including bladder perforations, hemorrhage, and mesh-related complications.^74^ A 2013 Cochrane Review included 10 trials and compared native tissue repair with non-absorbable synthetic transvaginal mesh repair.^75^ Women who underwent native tissue repair were more likely to have recurrent prolapse (RR 3.15) and report an increased awareness of their anterior vaginal wall than women who underwent a mesh procedure.^75^ A 2016 update to the Cochrane Review found that although repeat surgery for POP was lower after transvaginal mesh repair, repeat surgery for any pelvic floor disorder (i.e. POP, stress incontinence, or mesh exposure) was higher after mesh placement (RR 2.4).^76^ This was largely driven by the 8% repeat surgery for mesh exposure.^76^ Transvaginal mesh was also associated with higher rates of *de novo* stress incontinence and bladder injury.^76^ There are only a few trials which investigate the use of transvaginal mesh for the primary outcome of apical support, and many of these are limited by the use of trocar-based kits, which are no longer available. One multicenter, randomized controlled trial compared uterosacral ligament suspension with anterior/posterior colporrhaphy to vaginal mesh apical suspension using the PROLift (Ethicon, Sommerville, NJ, USA).^77^ The primary outcome measure was objective treatment success (POP-Q stage ≤1) at 12 months. The trial was halted early due to high rates of mesh erosion but showed no difference in objective outcome or recurrence of prolapse between native-tissue and mesh-augmented repairs.^77^ A more recent study evaluated the long-term outcomes (>5 years) of women undergoing the PROLift procedure.^78^ Of the 208 women who had a PROLift placed over 2 years at a single institution, only 70 women returned for 5-year postoperative questionnaires and 48 women consented to an exam. In this cohort of women, there was a trend towards improved apical support, but the study was underpowered to show a statistical difference.^78^ At 5 years 94% of the women did not have prolapse beyond the hymen, and all patients had improvement in the prolapse questionnaire scores.^78^ Two additional trials of transvaginal mesh for apical support evaluate a kit called the IVS Tunneler (Tyco Healthcare, Plaisir, France), which has been removed from the market due to high complication rates.^79^,^80^ Therefore, the role of transvaginal mesh for apical prolapse is currently still under investigation. Finally, two high-quality randomized controlled trials of transvaginal mesh placed in the posterior compartment agree that there is no difference in symptomatic outcomes, and possible worse anatomic outcomes with mesh repairs.^81^,^82^

## TO STAGE OR NOT TO STAGE?

Considering that the risk factors for prolapse and stress urinary incontinence (SUI) are similar, it is not surprising that many women suffer from both disorders. For women with bothersome SUI, it is reasonable to offer them surgical correction of their SUI at the time of prolapse repair. However, optimal management of women without SUI symptoms undergoing POP repair is more controversial. More than 40% of women without SUI symptoms will develop SUI after POP repair.^21^,^83^ For many women, this trade-off of symptoms is unacceptable, so a number of well-designed trials have attempted to identify who would benefit from prophylactic continence procedures at the time of prolapse repair.

As described above, the CARE trial was designed to assess whether the addition of a prophylactic Burch colposuspension to abdominal sacrocolpopexy decreases postoperative SUI in stress continent women.^21^ Three months after surgery, 24% of the women in the Burch group and 44% of the controls met one or more of the predefined criteria for stress incontinence without an increase in urgency or voiding dysfunction.^21^ The CARE trial was the first well-designed study demonstrating benefits of prophylactic continence procedures at the time of POP repair in stress continent women. However, by the time of publication, increasing data and clinical practice favored midurethral slings over Burch as a less invasive option for treating SUI, making these data less generalizable.

In 2012, a multicenter US study, the OPUS trial, was published, investigating the role of prophylactic retropubic midurethral sling at the time of vaginal POP repair for apical and/or anterior vaginal wall POP in stress continent women.^83^ Investigators randomized 337 women with stage II–IV POP without SUI symptoms undergoing vaginal prolapse surgery to receive a retropubic midurethral sling or sham incisions.^83^ One year after surgery, the rate of *de novo* SUI was lower (27% versus 43%) in women receiving a midurethral sling.^83^ The number needed to treat with a midurethral sling to prevent one case of urinary incontinence was 6; however, unlike Burch, midurethral sling was associated with several important adverse outcomes including increased surgical times and increased rates of incomplete bladder emptying, surgical bleeding, and bladder perforation. Seven additional women in the sham group underwent surgical treatment for SUI in the first year; however, four women underwent sling revision for voiding dysfunction, resulting in only three additional returns to the operating room in the sham group. Preoperative reduced cough stress test may be useful in predicting incontinence outcomes. Seventy-two percent of women with a positive reduced cough stress test had urinary incontinence compared to only 30% with a negative cough stress test.^83^ Unlike addition of a Burch colposuspension, addition of a prophylactic midurethral sling was associated with risks as well as benefits; therefore, surgeons should discuss the potential advantages and disadvantages with each woman so she can make the best surgical choice based on her goals and preferences.

## THE FUTURE

The quantity of high-quality comparative effectiveness data for the surgical management of POP in women has markedly increased over the last decade. We can now discuss risks and benefits of multiple surgical procedures and help each woman make a well-informed choice that is consistent with her values. Unfortunately, using current techniques we have not eliminated POP recurrence or reoperation, complications, or the onset of new pelvic floor symptoms. Comparative effectiveness trials looking at the role of uterine preservation and new mesh/graft material are essential. Some investigators are looking for novel graft materials derived from the extracellular matrix that can be inserted at the time of prolapse repair to promote site-specific functional tissue remodeling without the risk of erosion.^84^ Perhaps the future of prolapse management should focus on disease prevention and advancing understanding of pathophysiology of POP, including role of collagen, connective tissue, nerve and muscle injury, and regeneration. Increasingly, investigators are looking for genetic variations that predispose women to early-onset prolapse with the hope that these genes can be selected out in future generations.^85^ Until these novel pathways of prevention and treatment reach patient application, it is important that a surgeon managing pelvic organ prolapse understands the array of options available for treating prolapse and is able to guide a patient’s decision-making with the available evidence on outcomes and risks.

## Abbreviations

CARE: colpopexy and urinary reduction efforts
POP: pelvic organ prolapse
POP-Q: prolapse quantification system
SSLF: sacrospinous ligament fixation
SUI: stress urinary incontinence
TVL: total vaginal length
USLS: uterosacral ligament suspension

## References

[1] Swift SE, Tate SB, Nicholas J (2003). Correlation of symptoms with degree of pelvic organ support in a general population of women: what is pelvic organ prolapse?. Am J Obstet Gynecol.

[2] Bradley CS, Nygaard IE (2005). Vaginal wall descensus and pelvic floor symptoms in older women. Obstet Gynecol.

[3] Nygaard I, Barber MD, Burgio KL (2008). Prevalence of symptomatic pelvic floor disorders in US women. JAMA.

[4] Rortveit G, Brown JS, Thom DH, Van Den Eeden SK, Creasman JM, Subak LL (2007). Symptomatic pelvic organ prolapse: prevalence and risk factors in a population-based, racially diverse cohort. Obstet Gynecol.

[5] Tegerstedt G, Maehle-Schmidt M, Nyrén O, Hammarström M (2005). Prevalence of symptomatic pelvic organ prolapse in a Swedish population. Int Urogynecol J Pelvic Floor Dysfunct.

[6] Wu J, Matthews CA, Conover MM, Pate V, Jonsson Funk M (2014). Lifetime risk of stress incontinence or pelvic organ prolapse surgery. Obstet Gynecol.

[7] FDA (2011). Urogynecologic surgical mesh: update on the safety and effectiveness of transvaginal mesh placement for pelvic organ prolapse.

[8] Summers A, Winkel LA, Hussain HK, DeLancey JO (2006). The relationship between anterior and apical compartment support. Am J Obstet Gynecol.

[9] Rooney K, Kenton K, Mueller ER, FitzGerald MP, Brubaker L (2006). Advanced anterior vaginal wall prolapse is highly correlated with apical prolapse. Am J Obstet Gynecol.

[10] Chen L, Ashton-Miller JA, Hsu Y, DeLancey JO (2006). Interaction among apical support, levator ani impairment, and anterior vaginal wall prolapse. Obstet Gynecol.

[11] Bump RC, Mattiasson A, Bo K (1996). The standardization of terminology of female pelvic organ prolapse and pelvic floor dysfunction. Am J Obstet Gynecol.

[12] Hsu Y, Chen L, Summers A, Ashton-Miller JA, DeLancey JO (2008). Anterior vaginal wall length and degree of anterior compartment prolapse seen on dynamic MRI. Int Urogynecol J Pelvic Floor Dysfunct.

[13] Shull BL (1999). Pelvic organ prolapse: anterior, superior, and posterior vaginal segment defects. Am J Obstet Gynecol.

[14] Toozs-Hobson P, Boos K, Cardozo L (1998). Management of vaginal vault prolapse. Br J Obstet Gynaecol.

[15] Alas AN, Bresee C, Eilber K (2015). Measuring the quality of care provided to women with pelvic organ prolapse. Am J Obstet Gynecol.

[16] Stewart JR, Hamner JJ, Heit MH (2016). Thirty years of cystocele/rectocele repair in the United States. Female Pelvic Med Reconstr Surg.

[17] Northington GM, Hudson CO, Karp DR, Huber SA (2016). Concomitant apical suspensory procedures in women with anterior vaginal wall prolapse in the United States in 2011. Int Urogynecol J.

[18] Denman MA, Gregory WT, Boyles SH, Smith V, Edwards SR, Clark AL (2008). Reoperation 10 years after surgically managed pelvic organ prolapse and urinary incontinence. Am J Obstet Gynecol.

[19] Guiahi M, Kenton K, Brubaker L (2008). Sacrocolpopexy without concomitant posterior repair improves posterior compartment defects. Int Urogynecol J Pelvic Floor Dysfunct.

[20] Brubaker L, Cundiff G, Fine P (2003). A randomized trial of colpopexy and urinary reduction efforts (CARE): design and methods. Control Clin Trials.

[21] Brubaker L, Cundiff G, Fine P (2006). Abdominal sacrocolpopexy with Burch colposuspension to reduce urinary stress incontinence. N Engl J Med.

[22] Brubaker L, Nygaard I, Richter HE (2008). Two-year outcomes after sacrocolpopexy with and without burch to prevent stress urinary incontinence. Obstet Gynecol.

[23] Bradley CS, Nygaard IE, Brown MB (2007). Bowel symptoms in women 1 year after sacrocolpopexy. Am J Obstet Gynecol.

[24] Grimes CL, Lukacz ES, Gantz MG (2014). What happens to the posterior compartment and bowel symptoms after sacrocolpopexy? Evaluation of 5-year outcomes from E-CARE. Female Pelvic Med Reconstr Surg.

[25] Maldonado PA, Montoya TI, Carrick MC Posterior vaginal wall anatomy: implications for surgical repair.

[26] Dariane C, Moszkowicz D, Peschaud F (2016). Concepts of the rectovaginal septum: implications for function and surgery. Int Urogynecol J.

[27] Huebner M, Rall K, Brucker SY, Reisenauer C, Siegmann-Luz KC, DeLancey JO (2014). The rectovaginal septum: visible on magnetic resonance images of women with Mayer-Rokitansky-Küster-Hauser syndrome (Müllerian agenesis). Int Urogynecol J.

[28] Kenton K, Shott S, Brubaker L (1999). Outcome after rectovaginal fascia reattachment for rectocele repair. Am J Obstet Gynecol.

[29] Maher CF, Qatawneh AM, Baessler K, Schluter PJ (2004). Midline rectovaginal fascial plication for repair of rectocele and obstructed defecation. Obstet Gynecol.

[30] Bovbjerg VE, Trowbridge ER, Barber MD, Martirosian TE, Steers WD, Hullfish KL (2009). Patient-centered treatment goals for pelvic floor disorders: association with quality-of-life and patient satisfaction. Am J Obstet Gynecol.

[31] Pakbaz M, Persson M, Löfgren M, Mogren I (2010). ‘A hidden disorder until the pieces fall into place’ - a qualitative study of vaginal prolapse. BMC Womens Health.

[32] Frick AC, Barber MD, Paraiso MF, Ridgeway B, Jelovsek JE, Walters MD (2013). Attitudes toward hysterectomy in women undergoing evaluation for uterovaginal prolapse. Female Pelvic Med Reconstr Surg.

[33] Korbly NB, Kassis NC, Good MM (2013). Patient preferences for uterine preservation and hysterectomy in women with pelvic organ prolapse. Am J Obstet Gynecol.

[34] Moorman PG, Myers ER, Schildkraut JM, Iversen ES, Wang F, Warren N (2011). Effect of hysterectomy with ovarian preservation on ovarian function. Obstet Gynecol.

[35] Farquhar CM, Sadler L, Harvey SA, Stewart AW (2005). The association of hysterectomy and menopause: a prospective cohort study. BJOG.

[36] Gutman R, Maher C (2013). Uterine-preserving POP surgery. Int Urogynecol J.

[37] Detollenaere RJ, den Boon J, Stekelenburg J (2015). Sacrospinous hysteropexy versus vaginal hysterectomy with suspension of the uterosacral ligaments in women with uterine prolapse stage 2 or higher: multicentre randomised non-inferiority trial. BMJ.

[38] Maher CF, Carey MP, Murray CJ (2001). Laparoscopic suture hysteropexy for uterine prolapse. Obstet Gynecol.

[39] Roovers JWR, van der Vaarta CH, van der Bomb JG, Schagen van Leeuwenc JH, Scholtend PC, Heintza APM (2004). A randomised controlled trial comparing abdominal and vaginal prolapse surgery: effects on urogenital function. BJOG.

[40] Jefferis H, Price N, Jackson S (2017). Laparoscopic hysteropexy: 10 years’ experience. Int Urogynecol J.

[41] Gutman RE, Rardin CR, Sokol ER (2017). Vaginal and laparoscopic mesh hysteropexy for uterovaginal prolapse: a parallel cohort study. Am J Obstet Gynecol.

[42] Nager CW, Zyczynski H, Rogers RG (2016). The design of a randomized trial of vaginal surgery for uterovaginal prolapse: vaginal hysterectomy with native tissue vault suspension versus mesh hysteropexy suspension (The Study of Uterine Prolapse Procedures Randomized Trial). Female Pelvic Med Reconstr Surg.

[43] Altman D, Mikkola TS, Bek KM (2016). Pelvic organ prolapse repair using the Uphold™ Vaginal Support System: a 1-year multicenter study. Int Urogynecol J.

[44] Jirschele K, Seitz M, Zhou Y, Rosenblatt P, Culligan P, Sand P (2015). A multicenter, prospective trial to evaluate mesh-augmented sacrospinous hysteropexy for uterovaginal prolapse. Int Urogynecol J.

[45] Maher C, Baessler K, Glazener CM, Adams EJ, Hagen S (2004). Surgical management of pelvic organ prolapse in women. Cochrane Database Syst Rev.

[46] Siddiqui NY, Grimes C, Casiano ER (2015). Mesh sacrocolpopexy compared with native tissue vaginal repair: a systematic review and meta-analysis. Obstet Gynecol.

[47] Chan JK, Gardner AB, Taylor K (2015). Robotic versus laparoscopic versus open surgery in morbidly obese endometrial cancer patients - a comparative analysis of total charges and complication rates. Gynecol Oncol.

[48] Horvath P, Lange J, Bachmann R, Struller F, Königsrainer A, Zdichavsky M (2017). Comparison of clinical outcome of laparoscopic versus open appendectomy for complicated appendicitis. Surg Endosc.

[49] Freeman RM, Pantazis K, Thomson A (2013). A randomized controlled trial of abdominal versus laparoscopic sacrocolpopexy for the treatment of post-hysterectomy vaginal vault prolapse: LAS study. Int Urogynecol J.

[50] Kearney R, DeLancey JO (2003). Selecting suspension points and excising the vagina during Michigan four-wall sacrospinous suspension. Obstet Gynecol.

[51] Richter K, Albrich W (1981). Long-term results following fixation of the vagina on the sacrospinal ligament by the vaginal route. Am J Obstet Gynecol.

[52] Larson KA, Smith T, Berger MB (2013). Long-term patient satisfaction with michigan four-wall sacrospinous ligament suspension for prolapse. Obstet Gynecol.

[53] Shull BL, Bachofen C, Coates KW, Kuehl TJ (2000). A transvaginal approach to repair of apical and other associated sites of pelvic organ prolapse with uterosacral ligaments. Am J Obstet Gynecol.

[54] Silva WA, Pauls RN, Segal JL, Rooney CM, Kleeman SD, Karram MM (2006). Uterosacral ligament vault suspension: five-year outcomes. Obstet Gynecol.

[55] Barber MD, Brubaker L, Burgio KL, Eunice Kennedy Shriver National Institute of Child Health and Human Development. Pelvic Floor Disorders Network (2014). Comparison of 2 transvaginal surgical approaches and perioperative behavioral therapy for apical vaginal prolapse: the OPTIMAL randomized trial. JAMA.

[56] Paraiso MF, Jelovsek JE, Frick A (2011). Laparoscopic compared with robotic sacrocolpopexy for vaginal prolapse. Obstet Gynecol.

[57] Anger JT, Mueller ER, Tarnay C (2014). Robotic compared with laparoscopic sacrocolpopexy: a randomized controlled trial. Obstetr Gynecol.

[58] Mueller ER, Kenton K, Anger JT, Bresee C, Tarnay C (2016). Cosmetic appearance of port-site scars 1 year after laparoscopic versus robotic sacrocolpopexy: a supplementary study of the ACCESS Clinical Trial. J Minim Invasive Gynecol.

[59] Hudson C, Northington G, Lyles R, Karp D (2014). Outcomes of robotic sacrocolpopexy: a systematic review and meta-analysis. Female Pelvic Med Recon Surg.

[60] Olsen AL, Smith VJ, Bergstrom JO, Colling JC, Clark AL (1997). Epidemiology of surgically managed pelvic organ prolapse and urinary incontinence. Obstet Gynecol.

[61] Maher CF, Qatawneh AM, Dwyer PL, Carey MP, Cornish A, Schluter PJ (2004). Abdominal sacral colpopexy or vaginal sacrospinous colpopexy for vaginal vault prolapse: a prospective randomized study. Am J Obstet Gynecol.

[62] Benson JT, Lucente V, McClellan E (1996). Vaginal versus abdominal reconstructive surgery for the treatment of pelvic support defects: a prospective randomized study with long-term outcome evaluation. Am J Obstet Gynecol.

[63] Thompson PK, McCrery RJ, Lotze EC, Sangi-Haghpeykar H (2008). Vaginal prolapse surgery: comparing abdominal sacral colpopexy to uterosacral suspension. J Pelvic Med Surg.

[64] Cundiff GW, Varner E, Visco AG, Zyczynski HM, Nager CW, Norton PA (2008). Risk factors for mesh/suture erosion following sacral colpopexy. Am J Obstet Gynecol.

[65] Kohli N, Walsh PM, Roat TW, Karram MM (1998). Mesh erosion after abdominal sacrocolpopexy. Obstet Gynecol.

[66] Visco AG, Weidner AC, Barber MD (2001). Vaginal mesh erosion after abdominal sacral colpopexy. Am J Obstet Gynecol.

[67] Nygaard I, Brubaker L, Zyczynski HM (2013). Long-term outcomes following abdominal sacrocolpopexy for pelvic organ prolapse. JAMA.

[68] Stepanian AA, Miklos JR, Moore RD, Mattox TF (2008). Risk of mesh extrusion and other mesh-related complications after laparoscopic sacral colpopexy with or without concurrent laparoscopic-assisted vaginal hysterectomy: experience of 402 patients. J Minim Invasive Gynecol.

[69] Brizzolara S, Pillai-Allen A (2003). Risk of mesh erosion with sacral colpopexy and concurrent hysterectomy. Obstet Gynecol.

[70] Nosti PA, Lowman JK, Zollinger TW, Hale DS, Woodman PJ (2009). Risk of mesh erosion after abdominal sacral colpoperineopexy with concomitant hysterectomy. Am J Obstet Gynecol.

[71] Geller EJ, Parnell BA, Dunivan GC (2012). Robotic vs abdominal sacrocolpopexy: 44-month pelvic floor outcomes. Urology.

[72] Shepherd JP, Higdon HL, Stanford EJ, Mattox TF (2010). Effect of suture selection on the rate of suture or mesh erosion and surgery failure in abdominal sacrocolpopexy. Female Pelvic Med Reconstr Surg.

[73] U.S. Food and Drug Administration FDA public health notification: serious complications associated with transvaginal placement of surgical mesh in repair of pelvic organ prolapse and stress urinary incontinence.

[74] Altman D, Väyrynen T, Engh ME, Axelsen S, Falconer C, Nordic Transvaginal Mesh Group (2011). Anterior colporrhaphy versus transvaginal mesh for pelvic-organ prolapse. N Engl J Med.

[75] Maher C, Feiner B, Baessler K (2013). Surgical management of pelvic organ prolapse in women. Cochrane Database Syst Rev.

[76] Maher C, Feiner B, Baessler K, Christmann-Schmid C, Haya N, Marjoribanks J (2016). Transvaginal mesh or grafts compared with native tissue vaginal repair for vaginal prolapse. Cochrane Database of Syst Rev.

[77] Sokol AI, Iglesia CB, Kudish BI (2012). One-year objective and functional outcomes of a randomized clinical trial of vaginal mesh for prolapse. Am J Obstet Gynecol.

[78] Meyer I, McGwin G, Swain TA, Alvarez MD, Ellington DR, Richter HE (2016). Synthetic graft augmentation in vaginal prolapse surgery: long-term objective and subjective outcomes. J Minim Invasive Gynecol.

[79] de Tayrac R, Mathe ML, Bader G, Deffieux X, Fazel A, Fernandez H (2008). Infracoccygeal sacropexy or sacrospinous suspension for uterine or vaginal vault prolapse. Int J Gynaecol Obstet.

[80] Cosma S, Menato G, Preti M (2014). Advanced utero-vaginal prolapse and vaginal vault suspension: synthetic mesh vs native tissue repair. Arch Gynecol Obstet.

[81] Paraiso MF, Barber MD, Muir TW, Walters MD (2006). Rectocele repair: a randomized trial of three surgical techniques including graft augmentation. Am J Obstet Gynecol.

[82] Sung VW, Rardin CR, Raker CA, Lasala CA, Myers DL (2012). Porcine subintestinal submucosal graft augmenttation for rectocele repair: a randomized controlled trial. Obstet Gynecol.

[83] Wei JT, Nygaard I, Richter HE (2012). A midurethral sling to reduce incontinence after vaginal prolapse repair. N Engl J Med.

[84] Liang R, Knight K, Barone W (2017). Extracellular matrix regenerative graft attenuates the negative impact of polypropylene prolapse mesh on vagina in rhesus macaque. Am J Obstet Gynecol.

[85] Borazjani A, Kow N, Harris S, Ridgeway B, Damaser MS (2017). Transcriptional regulation of connective tissue metabolism genes in women with pelvic organ prolapse. Female Pelvic Med Reconstr Surg.

